# The use of cisplatin in patients after kidney transplantation with chronic renal insufficiency: Is the benefit higher than potential risks in therapy of non-seminomatous germ cell tumors?

**DOI:** 10.1097/MD.0000000000026381

**Published:** 2021-06-18

**Authors:** Tomas Pokrivcak, Radek Lakomy, Tomas Kazda, Alexandr Poprach, Pavel Fabian, Igor Kiss

**Affiliations:** a Department of Comprehensive Cancer Care, Masaryk Memorial Cancer Institute; b Department of Comprehensive Cancer Care, Faculty of Medicine, Masaryk University, Kamenice 5; c Department of Radiation Oncology, Masaryk Memorial Cancer Institute; d Department of Pathology, Masaryk Memorial Cancer Institute, Brno, Czech Republic.

**Keywords:** cisplatin, germ-cell tumor, nephrotoxicity, treatment

## Abstract

**Rationale:**

The use of cisplatin in patients with chronic kidney disease (CKD) is risky and depends on a number of factors. The optimal procedure in stage I of a non seminomatous germ cell tumor without proven lymphangioinvasion after orchiectomy is controversial and is the subject of a number of discussions due to the lack of randomized studies assessing individual treatment options. The adjuvant method of choice is surveillance or application of cisplatin-based chemotherapy with the risk of treatment related nephrotoxicity. Information about cisplatin safety in renal transplant patients is particularly limited. The aim of this paper is to share the experience with the application of adjuvant chemotherapy Bleomycin, Etoposide, Cisplatin (BEP) in high-risk patient with nonseminoma after kidney transplantation.

**Patient concerns:**

We report a case report of rare group of high-risk patient with non-seminomatous germ cell testicular tumor (NSGCT) after kidney transplantation before application of adjuvant chemotherapy BEP. Patient presented with month-long discomfort in the scrotal area. Previously, he was treated with chronic kidney disease based on chronic glomerulonephritis, which was treated with repeated kidney transplantation.

**Diagnosis:**

The ultrasound examination for a month-long discomfort in the scrotal area found a solid mass of the left testis. Radical inguinal orchiectomy confirmed NSGCT with the presence of lymphovascular invasion (LVI). Postoperative staging with computed tomography of the chest and abdomen did not show obvious dissemination of the disease.

**Interventions:**

Reducing original dose of chemotherapeutics according to the recommendations of the summary of product characteristics led to only a transient increase in creatinine levels.

**Outcomes:**

The 5-year risk of relapse in surveillance was reduced to around 3% by applying cisplatin-based chemotherapy.

**Lessons:**

Application of cisplatin-based chemotherapy is safe and effective in patients with CKD and in patients with a kidney transplant.

## 1 Introduction

The optimal procedure in stage I of a non seminomatous germ cell tumor without proven lymphangioinvasion after orchiectomy is controversial and is the subject of a number of discussions due to the lack of randomized studies assessing individual treatment options. The risk of tumor relapse in 5 years after orchiectomy is in the absence of lymphangioinvasion in “low risk” tumors around 15%.^[1]^ The adjuvant method of choice is surveillance or application of cisplatin-based chemotherapy. In the presence of lymphangioinvasion is tumor defined as a “high risk” and the probability of tumor relapse in 5 years after surgery is about 50%. The 5-year risk of relapse in surveillance can be reduced to around 3% by applying cisplatin-based chemotherapy (BEP—Bleomycin, Etoposide, Cisplatin).^[2]^ One of the limiting side effects of cisplatin is nephrotoxicity. The use of cisplatin in patients with chronic renal failure is risky and depends on a number of factors. Information about cisplatin safety in renal transplant patients is particularly limited. The aim of this paper is to share the experience with the application of adjuvant chemotherapy BEP in high-risk patient with nonseminoma after kidney transplantation.

## 2 Case report

**Figure 1:**
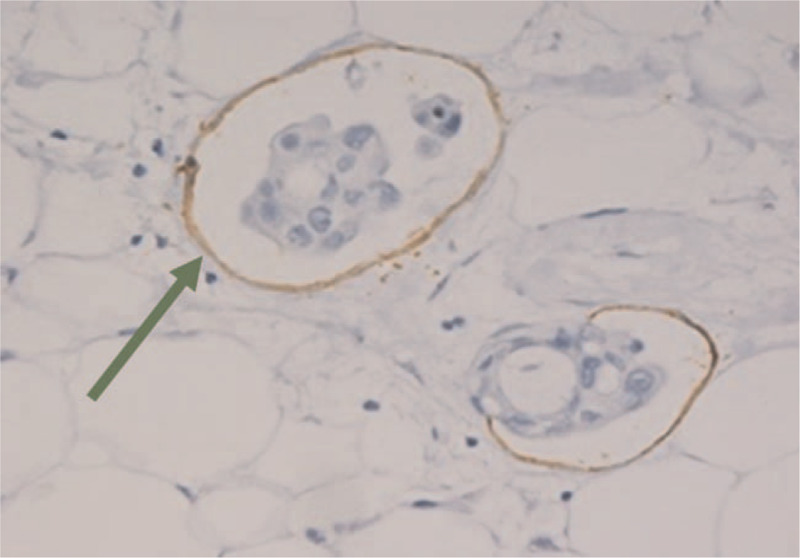
The presence of tumor cells within a lymphatic vessel lumen at 400× magnification.

A 58-year-old patient was referred to the urologist in January 2017 for a month-long discomfort in the scrotal area. The ultrasound examination found a solid mass of the left testis, which led to a radical inguinal orchiectomy on February 1, 2017. The examination by a pathologist showed a 20 mm tumor with histological findings of non-seminomatous germ cell testicular tumors (NSGCT). The dominant component was embryonal carcinoma with admixture of yolk sac tumor. The presence of lymphovascular invasion (LVI) was confirmed in the tumor (Fig. 1). Postoperative staging with computed tomography of the chest and abdomen did not show obvious dissemination of the disease. The level of tumor blood markers before and after surgery was without elevation. The definitive staging was closed according to the 8th TNM classification as pT2 N0 M0 S0—clinical stage IB. Due to the presence of LVI, the finding met the definition of “high risk” tumor. In March 2017, the patient was referred to the Masaryk Memorial Cancer Institute to consult further therapy. In addition to the tumor, the patient was also treated with chronic kidney disease (CKD) based on chronic glomerulonephritis, which was treated with repeated kidney transplantation. The first kidney transplantation was performed in December 2003. The second kidney transplantation—based on graft rejection—was performed in October 2008. Actual creatinine values ranged from 1.58 to 1.7 mg/dL and the graft function met the definition of CKD 3a-b.

**Figure 2:**
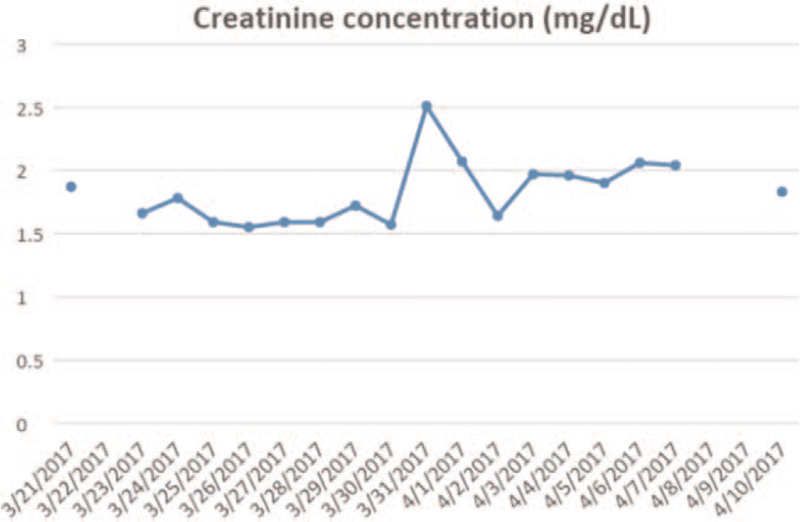
Time course of creatinine concentrations. Dates are in MM.DD.YYYY format.

The guidelines of the National Comprehensive Cancer Network and European Association of Urology recommend cisplatin-based adjuvant chemotherapy (BEP) in patients with stage I NSGCT with confirmed LVI.^[3,4]^ By applying of 1 series of BEP chemotherapy it is possible to reduce the risk of relapse in 5 years from 50% to 3.2%.^[2]^ Due to the CKD in the transplanted kidney, benefits and risks of chemotherapy were discussed with the patient, including the possibility of graft failure and the dialysis. In the second variant, the patient was also acquainted with the possibility of active surveillance. However, due to the presence of a high risk tumor, there was a risk of relapse of up to 50% during the first 5 years after orchiectomy. In case of dissemination, curative chemotherapy with ≥3 series of chemotherapy would be then indicated with uncertain results and higher toxicity than in adjuvant administration. The functionality of the transplanted kidney and the time from transplantation to the time of diagnosis (9 years) also played an important role in the shared decision-making process. In this context, there was a risk of dialysis for 2 to 3 years, followed by a very limited possibility of using chemotherapy at that time. These options have been repeatedly discussed with the patient. Based on the information provided, patient agreed to the application of 1 series of adjuvant chemotherapy BEP. According to the recommendations of the Summary of product characteristics, the dose of individual chemotherapeutics was reduced to 75% of the original dose.^[5]^ Due to the risk of nephrotoxicity, chemotherapy was administered through the intensive care unit from March 22, 2017 to March 26, 2017 with careful monitoring of fluid balance and creatinine levels. On day 9, the patient developed fever above 38°. The control laboratory showed grade 4 neutropenia and an elevated level of CRP. Due to the presence of febrile neutropenia, therapy with broad-spectrum antibiotics was initiated. Following administration of 5 granulocyte colony stimulating factor, neutrophil levels normalized. Other forms of hematological toxicity included grade 4 asymptomatic thrombocytopenia. Diuresis was sufficient, with creatinine fluctuations from 1.58 to 2.51 mg/dL, Fig. 2. The further course then proceeded without complications and the patient was subsequently discharged in a stable state and with satisfactory laboratory parameters for home treatment. Relapse of the disease has not been proven so far in the dispensary. The last computed tomography examination of the abdomen in September 2020 was without evidence of metastases. At regular nephrology examinations, the transplanted kidney has a stable function, without the need to include the patient in a dialysis program. Informed consent was obtained from the patient for the purpose of research and presentation of data in anonymous form.

## 3 Discussion

According to the Surveillance, Epidemiology, and End Results database, >67% of testicular tumors are diagnosed in stage I and >60% have >1 histological component.^[6]^ The optimal procedure for stage I NSGCT is inconsistent and depends on the presence of risk factors, most importantly by presence of LVI.^[7]^ The management after orchiectomy includes surveillance, cisplatin-based chemotherapy (BEP), and retroperitoneal lymph node dissection. Among chemotherapeutics with potentially lower nephrotoxicity, the inferiority of the carboplatin-Carboplatin, Etoposide, Bleomycin (CEB) versus BEP regimen has been previously demonstrated in randomized trials.^[8,9]^ In the Horwich study, patients received either 4 series of BEP or 4 series of CEB. Failure after BEP chemotherapy occurred in 30 patients compared with 79 cases after CEB (log-rank *X*^2^ = 6.9; *P* < .001). The most important risk factor is the presence of LVI, which is detected in about 1/3 of patients.^[10]^ In the presence of LVI, the tumor is defined as a “high risk” and the risk of relapse in 5 years after surgery is about 50%. In the absence of LVI, it is a “low risk” tumor and the risk of relapse decreases to 15% to 20%.^[1]^ The current recommendation of the NCCCN based on data from the prospective study SWENOTECA in low-risk tumors (LVI-) leaves the choice between surveillance and 1 series of BEP. The 5-year risk of relapse in surveillance can be reduced from 15% to 1.6% by applying 1× BEP. For high risk tumors (LVI +), the application of 1 series of BEP is recommend which can reduce the risk of relapse to 3.2%.^[2]^ Original data from Cullen et al^[11]^ from 1996 without stratification of patients according to risk factors show a relapse risk of 2.7% after administration of 2 series of adjuvant BEP chemotherapy. A number of studies have confirmed a direct link between the total dose of chemotherapy administered and the increased incidence of late adverse events.^[12–14]^ However, in most studies, the incidence of adverse reactions was reported following the curative chemotherapy—≥3 cisplatin-based series. Typical adverse reactions associated with cisplatin include kidney damage, high emetogenic potential, peripheral neuropathy, ototoxicity, and hematological toxicity potentiated by other chemotherapeutic agents. Due to the predominant renal excretion of cisplatin, the concentration in tubular epithelial cells is 5 times higher than in blood. An early clinical use of cisplatin has demonstrated the occurrence of acute renal failure in direct response to cumulative dose.^[15]^ The current use of cisplatin in regimens involving pre and posthydration with careful monitoring of diuresis in the treatment of testicular tumors is associated with renal impairment in about 20% to 30% of patients. The renal function generally recovers 2 to 4 weeks after treatment.^[16]^ In our patient, despite the limited lifespan of the transplanted kidney, dialysis has not been indicated for 11 years. The efficacy and safety of cisplatin-based chemotherapy have been demonstrated in the past in a number of case reports in renal transplant patients.^[17–21]^ It, however, included cases of patients with disseminated NSGCT where the absence of chemotherapy led to disease progression.

In summary, based on our experience and presented case reports, it can be assumed that the application of cisplatin-based chemotherapy is safe and effective in CKD and in patients with a kidney transplant. An important part of the therapy is careful fluid balance and control of the internal environment. The key part is sharing the decision-making process with the patient.

## Author contributions

**Conceptualization:** Tomas Pokrivcak, Radek Lakomy, Pavel Fabian, Igor Kiss.

**Data curation:** Tomas Pokrivcak.

**Funding acquisition:** Igor Kiss.

**Investigation:** Radek Lakomy, Pavel Fabian.

**Methodology:** Tomas Pokrivcak, Pavel Fabian.

**Resources:** Tomas Kazda.

**Supervision:** Alexandr Poprach.

**Writing – original draft:** Tomas Pokrivcak, Radek Lakomy, Tomas Kazda.

**Writing – review & editing:** Tomas Pokrivcak, Radek Lakomy, Tomas Kazda, Alexandr Poprach, Igor Kiss.
